# Conversion of Continuous-Valued Deep Networks to Efficient Event-Driven Networks for Image Classification

**DOI:** 10.3389/fnins.2017.00682

**Published:** 2017-12-07

**Authors:** Bodo Rueckauer, Iulia-Alexandra Lungu, Yuhuang Hu, Michael Pfeiffer, Shih-Chii Liu

**Affiliations:** ^1^Institute of Neuroinformatics, University of Zurich and ETH Zurich, Zurich, Switzerland; ^2^Bosch Center for Artificial Intelligence, Renningen, Germany

**Keywords:** artificial neural network, spiking neural network, deep learning, object classification, deep networks, spiking network conversion

## Abstract

*Spiking* neural networks (SNNs) can potentially offer an efficient way of doing inference because the neurons in the networks are sparsely activated and computations are event-driven. Previous work showed that simple continuous-valued deep Convolutional Neural Networks (CNNs) can be converted into accurate spiking equivalents. These networks did not include certain common operations such as max-pooling, softmax, batch-normalization and Inception-modules. This paper presents spiking equivalents of these operations therefore allowing conversion of nearly arbitrary CNN architectures. We show conversion of popular CNN architectures, including VGG-16 and Inception-v3, into SNNs that produce the best results reported to date on MNIST, CIFAR-10 and the challenging ImageNet dataset. SNNs can trade off classification error rate against the number of available operations whereas deep continuous-valued neural networks require a fixed number of operations to achieve their classification error rate. From the examples of LeNet for MNIST and BinaryNet for CIFAR-10, we show that with an increase in error rate of a few percentage points, the SNNs can achieve more than 2x reductions in operations compared to the original CNNs. This highlights the potential of SNNs in particular when deployed on power-efficient neuromorphic spiking neuron chips, for use in embedded applications.

## 1. Introduction

Deep Artificial Neural Network (ANN) architectures such as GoogLeNet (Szegedy et al., 2015) and VGG-16 (Simonyan and Zisserman, 2014) have successfully pushed the state-of-the-art classification error rates to new levels on challenging computer vision benchmarks like ImageNet (Russakovsky et al., 2015). Inference in such very large networks, i.e., classification of an ImageNet frame, requires substantial computational and energy costs, thus limiting their use in mobile and embedded applications.

Recent work have shown that the event-based mode of operation in SNNs is particularly attractive for reducing the latency and computational load of deep neural networks (Farabet et al., 2012; O'Connor et al., 2013; Neil et al., 2016; Zambrano and Bohte, 2016). Deep SNNs can be queried for results already after the first output spike is produced, unlike ANNs where the result is available only after all layers have been completely processed (Diehl et al., 2015). SNNs are also naturally suited to process input from event-based sensors (Posch et al., 2014; Liu et al., 2015), but even in classical frame-based machine vision applications such as object recognition or detection, they have been shown to be accurate, fast, and efficient, in particular when implemented on neuromorphic hardware platforms (Neil and Liu, 2014; Stromatias et al., 2015; Esser et al., 2016). SNNs could thus play an important role in supporting, or in some cases replacing deep ANNs in tasks where fast and efficient classification in real-time is crucial, such as detection of objects in larger and moving scenes, tracking tasks, or activity recognition (Hu et al., 2016).

Multi-layered spiking networks have been implemented on digital commodity platforms such as FPGAs (Neil and Liu, 2014; Gokhale et al., 2014), but spiking networks with more than tens of thousands of neurons can be implemented on large-scale neuromorphic spiking platforms such as TrueNorth (Benjamin et al., 2014; Merolla et al., 2014) and SpiNNaker (Furber et al., 2014). Recent demonstrations with TrueNorth (Esser et al., 2016) show that CNNs of over a million neurons can be implemented on a set of chips with a power dissipation of only a few hundred mW. Given the recent successes of deep networks, it would be advantageous if spiking forms of deep ANN architectures such as VGG-16 can be implemented on these power-efficient platforms while still producing good error rates. This would allow the deployment of deep spiking networks in combination with an event-based sensor for real-world applications (Orchard et al., 2015a; Serrano-Gotarredona et al., 2015; Kiselev et al., 2016).

In order to bridge the gap between Deep Learning continuous-valued networks and neuromorphic spiking networks, it is necessary to develop methods that yield deep *Spiking* Neural Networks (SNNs) with equivalent error rates as their continuous-valued counterparts. Successful approaches include direct training of SNNs using backpropagation (Lee et al., 2016), the SNN classifier layers using stochastic gradient descent (Stromatias et al., 2017), or modifying the transfer function of the ANNs during training so that the network parameters can be mapped better to the SNN (O'Connor et al., 2013; Esser et al., 2015; Hunsberger and Eliasmith, 2016). The largest architecture trained by Hunsberger and Eliasmith (2016) in this way is based on AlexNet (Krizhevsky et al., 2012). While the results are promising, these novel methods have yet to mature to the state where training spiking architectures of the size of VGG-16 becomes possible, and the same state-of-the-art error rate as the equivalent ANN is achieved.

A more straightforward approach is to take the parameters of a pre-trained ANN and to map them to an equivalent-accurate SNN. Early studies on ANN-to-SNN conversion began with the work of Perez-Carrasco et al. (2013), where CNN units were translated into biologically inspired spiking units with leaks and refractory periods, aiming for processing inputs from event-based sensors. Cao et al. (2015) suggested a close link between the transfer function of a spiking neuron, i.e., the relation between input current and output firing frequency to the activation of a rectified linear unit (ReLU), which is nowadays the standard model for the neurons in ANNs. They report good performance error rates on conventional computer vision benchmarks, converting a class of CNNs that was restricted to having zero bias and only average-pooling layers. Their method was improved by Diehl et al. (2015), who achieved nearly loss-less conversion of ANNs for the MNIST (LeCun et al., 1998) classification task by using a *weight normalization* scheme. This technique rescales the weights to avoid approximation errors in SNNs due to either excessive or too little firing of the neurons. Hunsberger and Eliasmith (2016) introduced a conversion method where noise injection during training improves the robustness to approximation errors of the SNN with more realistic biological neuron models. Esser et al. (2016) demonstrated an approach that optimized CNNs for the TrueNorth platform which has binary weights and restricted connectivity. Zambrano and Bohte (2016) have developed a conversion method using spiking neurons that adapt their firing threshold to reduce the number of spikes needed to encode information.

These approaches achieve very good results on MNIST, but the SNN results are below state-of-the-art ANN results when scaling up to networks that can solve CIFAR-10 (Krizhevsky, 2009). One reason is that SNN implementations of many operators that are crucial for improved ANN error rate, such as max-pooling layers, softmax activation functions, and batch-normalization, are non-existent, and thus SNNs can only approximately match the inference of an ANN. As a consequence, none of the previously proposed conversion approaches are general enough for full automatic conversion of arbitrary pre-trained ANNs taken from a Deep-Learning model zoo available, for example, in Caffe^1^.

In this work, we address some important shortcomings of existing ANN-to-SNN conversion methods. Through mathematical analysis of the approximation of the output firing rate of a spiking neuron to the equivalent analog activation value, we were able to derive a theoretical measure of the error introduced in the previous conversion process. On the basis of this novel theory, we propose modifications to the spiking neuron model that significantly improve the performance of deep SNNs. By developing spiking implementations of max-pooling layers, softmax activation, neuron biases, and batch normalization (Ioffe and Szegedy, 2015), we extend the suite of CNNs that can be converted. In particular, we demonstrate for the first time that GoogLeNet Inception-V3 can be converted to an equivalent-accurate SNN. Further, we show that the conversion to spiking networks is synergistic with ANN network compression techniques such as parameter quantization and the use of low-precision activations.

To automate the process of transforming a pre-trained ANN into an SNN, we developed an SNN-conversion toolbox that is able to transform models written in Keras (Chollet, 2015), Lasagne and Caffe, and offers built-in simulation tools for evaluation of the spiking model. Alternatively, the converted SNN can be exported for use in spiking simulators like pyNN or Brian2. The documentation and source code is publicly available online^2^.

The remainder of the paper is organized as follows: section 2.1 outlines the conversion theory and section 2.2 presents the methods for implementing the different features of a CNN. The work in these two sections is extended from earlier work in Rueckauer et al. (2016). section 3 presents the conversion results of networks tested on the MNIST, CIFAR-10, and ImageNet datasets.

## 2. Methods

### 2.1. Theory for conversion of ANNs into SNNs

The basic principle of converting ANNs into SNNs is that firing rates of spiking neurons should match the graded activations of analog neurons. Cao et al. (2015) first suggested a mechanism for converting (ReLU) activations, but a theoretical groundwork for this principle was lacking. Here we present an analytical explanation for the approximation, and on its basis we are able to derive a simple modification of the reset mechanism following a spike, which turns each SNN neuron into an unbiased approximator of the target function (Rueckauer et al., 2016).

We assume here a one-to-one correspondence between an ANN unit and a SNN neuron, even though it is also possible to represent each ANN unit by a population of spiking neurons. For a network with *L* layers let **W**^*l*^, *l* ∈ {1, …, *L*} denote the weight matrix connecting units in layer *l* − 1 to layer *l*, with biases **b**^*l*^. The number of units in each layer is *M*^*l*^. The ReLU activation of the continuous-valued neuron *i* in layer *l* is computed as:

(1) $$\begin{array}{l} a_{i}^{l}:=\max\left(0,\sum_{j=1}^{M^{l-1}}W_{ij}^{l}a_{j}^{l-1}+b_{i}^{l}\right), \end{array}$$

starting with a^0^ = x, where x is the input, normalized so that each *x*_*i*_ ∈ [0, 1]^3^. Each SNN neuron has a membrane potential $V_{i}^{l}\left(t\right)$, which integrates its input current at every time step:

(2) $$\begin{array}{l} z_{i}^{l}(t):=V_{\text{thr}}\left(\sum_{j=1}^{M^{l-1}}W_{ij}^{l}\Theta_{t,j}^{l-1}+b_{i}^{l}\right), \end{array}$$

where *V*_thr_ is the threshold and $\Theta_{t,i}^{l}$ is a step function indicating the occurrence of a spike at time *t*:

(3) $$\begin{array}{l} \Theta_{t,i}^{l}:=\Theta (V_{i}^{l}(t-1)+z_{i}^{l}(t)-V_{\text{thr}}),\text{ with }\Theta (x)=\{\begin{array}{ll} 1 & \text{ if }x\geq 0\\ 0 & \text{ else.} \end{array} \end{array}$$

Every input pattern is presented for *T* time steps, with time step size Δ*t* ∈ ℝ^+^. The highest firing rate supported by a time stepped simulator is given by the inverse time resolution *r*_max_: = 1/Δ*t*. Input rates to the first layer are proportional to the constant pixel intensities or RGB image values. We can compute the firing rate of each SNN neuron *i* as $r_{i}^{l}\left(t\right):=N_{i}^{l}\left(t\right)/t$, where $N_{i}^{l}\left(t\right):=\overset{t}{\underset{t^{\prime }=1}{\sum }}\Theta_{t^{\prime },i}^{l}$ is the number of spikes generated.

The principle of the ANN-to-SNN conversion method as introduced in Cao et al. (2015), Diehl et al. (2015), postulates that the firing rate of a neuron $r_{i}^{l}$ correlates with its original ANN activation $a_{i}^{l}$ in (1). In the following, we introduce a membrane equation for the spiking neurons to formalize a concrete relationship $r_{i}^{l}\left(t\right)\propto a_{i}^{l}$.

#### 2.1.1. Membrane equation

The spiking neuron integrates inputs $z_{i}^{l}\left(t\right)$ until the membrane potential $V_{i}^{l}\left(t\right)$ exceeds a threshold $V_{\text{thr}}\in {\mathbb{R}}^{+}$ and a spike is generated. Once the spike is generated, the membrane potential is reset. We discuss next two types of reset: *reset to zero*, used e.g., in Diehl et al. (2015), always sets the membrane potential back to a baseline, typically zero. *Reset by subtraction*, or “linear reset mode” in Diehl et al. (2016); Cassidy et al. (2013), subtracts the threshold *V*_thr_ from the membrane potential at the time when it exceeds the threshold:

(4a,4b) $$\begin{array}{l} V_{i}^{l}(t)=\{\begin{array}{ll} \left(V_{i}^{l}(t-1)+z_{i}^{l}(t)\right)\;\left(1-\Theta_{t,i}^{l}\right) & \text{reset to zero}\\ V_{i}^{l}(t-1)+z_{i}^{l}(t)\;-V_{\text{thr}}\Theta_{t,i}^{l} & \text{reset by subtraction}. \end{array} \end{array}$$

From these membrane equations, we can derive slightly different approximation properties for the two reset mechanisms. In this section we analyze the first hidden layer and expand the argument in section 2.1.2 to higher layers. We assume that the input currents $z_{i}^{1}>0$ remain constant over time, and justify this assumption in section 2.2.4. The input to first-layer neurons (2) is then related to the ANN activations (1) via $z_{i}^{1}=V_{\text{thr}}a_{i}^{1}$. In order to relate these ANN activations to the SNN spike rates, we merely have to average the membrane Equations (4a) and (4b) over the simulation time. The detailed calculations are given in the Supplementary Material; the resulting rates are obtained as

(5a,5b) $$\begin{array}{l} r_{i}^{1}(t)=\{\begin{array}{ll} a_{i}^{1}r_{\text{max}}\cdot \frac{V_{\text{thr}}}{V_{\text{thr}}+\epsilon_{i}^{1}}-\frac{V_{i}^{1}(t)}{t\cdot (V_{\text{thr}}+\epsilon_{i}^{l})} & \text{reset to zero}\\ a_{i}^{1}r_{\text{max}}\;-\frac{V_{i}^{1}(t)}{t\cdot V_{\text{thr}}} & \text{reset by subtraction}. \end{array} \end{array}$$

As expected, the spike rates are proportional to the ANN activations $a_{i}^{1}$, but reduced by an additive approximation error term, and in case of *reset to zero* an additional multiplicative error term. In the *reset to zero* case, with constant input, there is always a constant number of time steps $n_{i}^{1}$ between spikes of the same neuron *i*, and the threshold will always be exceeded by the same constant amount $\epsilon_{i}^{1}=V_{i}^{1}\left(n_{i}^{1}\right)-V_{\text{thr}}=n_{i}^{1}\cdot z_{i}^{1}-V_{\text{thr}}\geq 0$. This residual charge $\epsilon_{i}^{1}$ is discarded at reset, which results in a reduced firing rate and thereby loss of information. For shallow networks and small datasets such as MNIST, this error seems to be a minor problem but we have found that an accumulation of approximation errors in deeper layers degrades the classification error rate. We also see from Equation (5a) that a larger *V*_thr_ and smaller inputs improve the approximation at the expense of longer integration times. Using the definition $\left(n_{i}^{1}-1\right)z_{i}^{1}<V_{\text{thr}}\leq n_{i}^{1}z_{i}^{1}$ for $n_{i}^{1}$ and $\epsilon_{i}^{1}=n_{i}^{1}z_{i}^{1}-V_{\text{thr}}$ for $\epsilon_{i}^{1}$, we find that the approximation error is limited from above by the magnitude of the input $z_{i}^{1}$. This insight further explains why the weight normalization scheme of Diehl et al. (2015) improves performance in the reset-to-zero case: By guaranteeing that the ANN activations $a_{i}^{1}$ are too low to drive a neuron in the SNN above *V*_thr_ within a single time step, we can keep $z_{i}^{1}=V_{\text{thr}}a_{i}^{1}$ and thereby $\epsilon_{i}^{l}$ low. Another obvious way of improving the approximation is to reduce the simulation time step, but this comes at the cost of increased computational effort.

A simple switch to the *reset by subtraction* mechanism improves the approximation, and makes the conversion scheme suitable also for deeper networks. The excess charge ϵ is not discarded at reset and can be used for the next spike generation. Accordingly, the error term due to ϵ does not appear in Equation (5b). Instead, the firing rate estimate in the first hidden layer converges to its target value $a_{i}^{1}\cdot r_{\text{max}}$; the only approximation error due to the discrete sampling vanishes over time. We validate by simulations in section 3.1 that this mechanism indeed leads to more accurate approximations of the underlying ANN than the methods proposed in Cao et al. (2015), Diehl et al. (2015), in particular for larger networks.

#### 2.1.2. Firing rates in higher layers

The previous results were based on the assumption that the neuron receives a constant input *z* over the simulation time. When neurons in the hidden layers are spiking, this condition only holds for the first hidden layer and for inputs in the form of analog currents instead of irregular spike trains. In the *reset-by-subtraction* case, we can derive analytically how the approximation error propagates through the deeper layers of the network. For this, we insert the expression for SNN input $z_{i}^{l}$ from Equation (2) into the membrane Equation (4b) for *l* > 1, average $V_{i}^{l}\left(t\right)$ over the simulation time, and solve for the firing rate $r_{i}^{l}\left(t\right)$. This yields:

(6) $$\begin{array}{l} r_{i}^{l}\left(t\right)=\overset{M^{l-1}}{\underset{j=1}{\sum }}W_{ij}^{l}r_{j}^{l-1}\left(t\right)+r_{\text{max}}b_{i}^{l}-\frac{V_{i}^{l}\left(t\right)}{t\cdot V_{\text{thr}}}. \end{array}$$

This equation states that the firing rate of a neuron in layer *l* is given by the weighted sum of the firing rates of the previous layer, minus the time-decaying approximation error described in Equation (5b). This relationship implies that each layer computes a weighted sum of the approximation errors of earlier layers, and adds its own approximation error. The recursive expression Equation (6) can be solved iteratively by inserting the expression for the previous layer rates, starting with the known rates of the first layer Equation (5b):

(7) $$\begin{array}{l} r_{i}^{l}=a_{i}^{l}r_{\text{max}}-\Delta V_{i_{l}}^{l}-\overset{M^{l-1}}{\underset{i_{l-1}=1}{\sum }}W_{i_{l}i_{l-1}}^{l}\Delta V_{i_{l-1}}^{l-1}-\cdots \\ -\;\overset{M^{l-1}}{\underset{i_{l-1}=1}{\sum }}W_{i_{l}i_{l-1}}^{l}\cdots \overset{M^{1}}{\underset{i_{1}=1}{\sum }}W_{i_{2}i_{1}}^{2}\Delta V_{i_{1}}^{1} \end{array}$$

with $\Delta V_{i}^{l}:=V_{i}^{l}\left(t\right)/\left(t\cdot V_{\text{thr}}\right)$. Thus, a neuron *i* in layer *l* receives an input spike train with a slightly lower spike rate, reduced according to the quantization error Δ*V* of previous layer neurons. These errors accumulate for higher layers, which explains why it takes longer to achieve high correlations of ANN activations, and why SNN firing rates deteriorate in higher layers.

### 2.2. Spiking implementations of ANN operators

In this section we introduce new methods that improve the classification error rate of deep SNNs (Rueckauer et al., 2016). These methods either allow the conversion of a wider ranger of ANNs, or reduce the approximation errors in the SNN.

#### 2.2.1. Converting biases

Biases are standard in ANNs, but were explicitly excluded by previous conversion methods for SNNs. In a spiking network, a bias can simply be implemented with a constant input current of equal sign as the bias. Alternatively, one could present the bias with an external spike input of constant rate proportional to the ANN bias, as proposed in Neftci et al. (2014), though then one may have to invert the sign of spikes to account for negative biases. The theory in section 2.1 can be applied to the case of neurons with biases, and the following section 2.2.2 shows how parameter normalization can be applied to biases as well.

#### 2.2.2. Parameter normalization

One source of approximation errors is that in time-stepped simulations of SNNs, the neurons are restricted to a firing rate range of [0, *r*_max_], whereas ANNs typically do not have such constraints. Weight normalization is introduced by Diehl et al. (2015) as a means to avoid approximation errors due to too low or too high firing. This work showed significant improvement of the performance of converted SNNs by using a *data-based weight normalization* mechanism. We extend this method to the case of neurons with biases and suggest a method that makes the normalization process more robust to outliers.

##### 2.2.2.1. Normalization with biases

The *data-based weight normalization* mechanism is based on the linearity of the ReLU unit used for ANNs. It can simply be extended to biases by linearly rescaling all weights and biases such that the ANN activation *a* [as computed in Equation (1)] is smaller than 1 for all training examples. In order to preserve the information encoded within a layer, the parameters of a layer need to be scaled jointly. Denoting the maximum ReLU activation in layer *l* as λ^*l*^ = max[**a**^*l*^], then weights **W**^*l*^ and biases **b**^*l*^ are normalized to ${\text{W}}^{l}\to {\text{W}}^{l}\frac{\lambda^{l-1}}{\lambda^{l}}$ and **b**^*l*^ → **b**^*l*^/λ^*l*^.

##### 2.2.2.2. Robust normalization

Although weight normalization avoids firing rate saturation in SNNs, it might result in very low firing rates, thereby increasing the latency until information reaches the higher layers. We refer to the algorithm described in the previous paragraph as “max-norm,” because the normalization factor λ^*l*^ was set to the maximum ANN activation within a layer, where the activations are computed using a large subset of the training data. This is a very conservative approach, which ensures that the SNN firing rates will most likely not exceed the maximum firing rate. The drawback is that this procedure is prone to be influenced by singular outlier samples that lead to very high activations, while for the majority of the remaining samples, the firing rates will remain considerably below the maximum rate.

**Figure 1:**
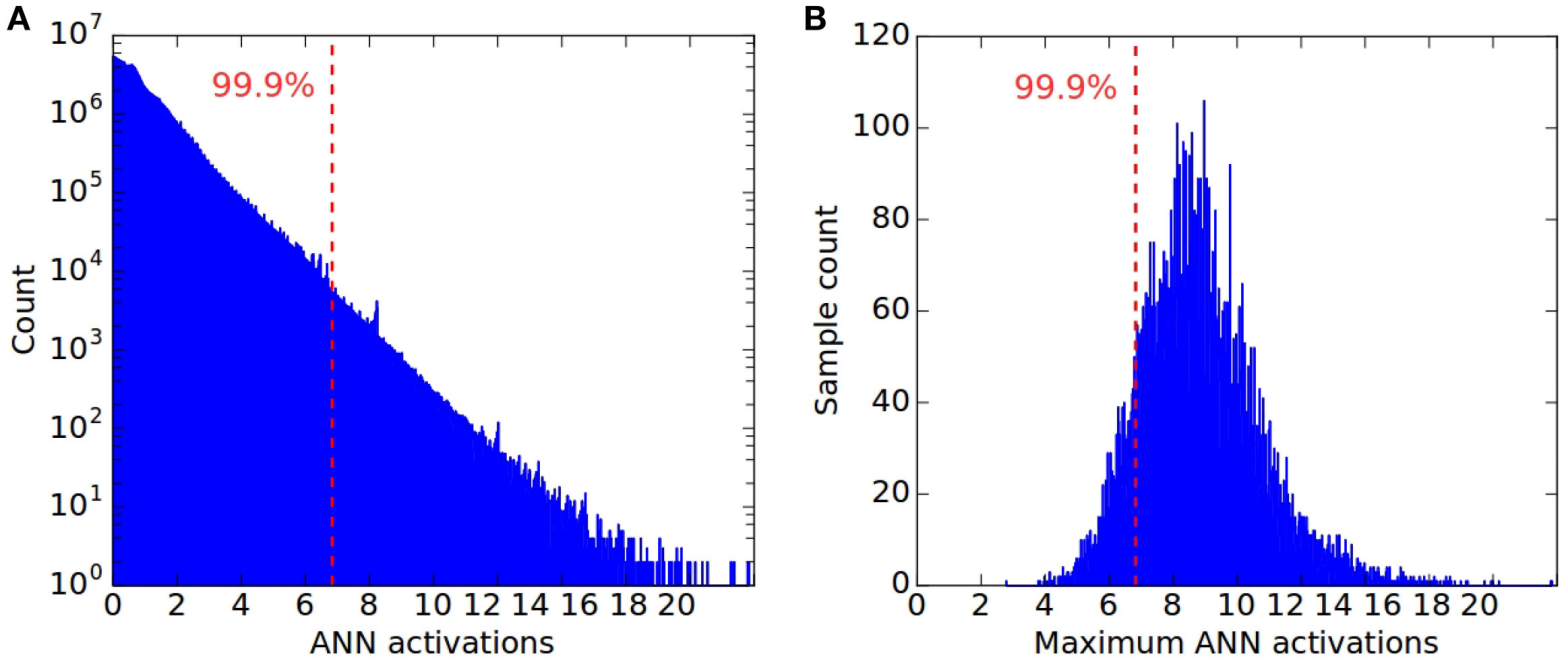
Distribution of all non-zero activations in the first convolution layer of a CNN, for 16666 CIFAR10 samples, and plotted in log-scale. The dashed line in both plots indicates the 99.9th percentile of all ReLU activations across the dataset, corresponding to a normalization scale λ = 6.83. This is more than three times less than the overall maximum of λ_*max*_ = 23.16. **(B)** Distribution of maximum ReLU activations for the same 16666 CIFAR10 samples. For most samples their maximum activation is far from λ_*max*_. **(A)** ANN activations; **(B)** Maximum ANN activation.

Such outliers are not uncommon, as shown in Figure 1A, which plots the log-scale distribution of all non-zero activations in the first convolution layer for 16,666 CIFAR10 samples. The maximum observed activation is more than three times higher than the 99.9th percentile. Figure 1B shows the distribution of the highest activations across the 16,666 samples for all ANN units in the same layer, revealing a large variance across the dataset, and a peak that is far away from the absolute maximum. This distribution explains why normalizing by the maximum can result in a potentially poor classification performance of the SNN. For the vast majority of input samples, even the maximum activation of units within a layer will lie far below the chosen normalization scale leading to insufficient firing within the layer to drive higher layers and subsequently worse classification results.

We propose a more robust alternative where we set λ^*l*^ to the *p*-th percentile of the total activity distribution of layer *l*^4^. This choice discards extreme outliers, and increases SNN firing rates for a larger fraction of samples. The potential drawback is that a small percentage of neurons will saturate, so choosing the normalization scale involves a trade-off between saturation and insufficient firing. In the following, we refer to the percentile *p* as the “normalization scale,” and note that the “max-norm” method is recovered as the special case *p* = 100. Typical values for *p* that perform well are in the range [99.0, 99.999]. In general, saturation of a small fraction of neurons leads to a lower degradation of the network classification error rate compared to the case of having spike rates that are too low. This method can be combined with batch-normalization (BN) used during ANN training (Ioffe and Szegedy, 2015), which normalizes the activations in each layer and therefore produces fewer extreme outliers.

#### 2.2.3. Conversion of batch-normalization layers

Batch-normalization reduces internal covariate shift in ANNs and thereby speeds up the training process. BN introduces additional layers where affine transformations of inputs are performed in order to achieve zero-mean and unit variance. An input *x* is transformed into $\text{BN}\left[x\right]=\frac{\gamma }{\sigma }\left(x-\mu \right)+\beta$, where mean μ, variance σ, and the two learned parameters β and γ are all obtained during training as described in Ioffe and Szegedy (2015). After training, these transformations can be integrated into the weight vectors, thereby preserving the effect of BN, but eliminating the need to compute the normalization repeatedly for each sample during inference. Specifically, we set ${\tilde{W}}_{ij}^{l}=\frac{\gamma_{i}^{l}}{\sigma_{i}^{l}}W_{ij}^{l}$ and ${\tilde{b}}_{i}^{l}=\frac{\gamma_{i}^{l}}{\sigma_{i}^{l}}\left(b_{i}^{l}-\mu_{i}^{l}\right)+\beta_{i}^{l}$. This makes it simple to convert BN layers into SNNs, because after transforming the weights of the preceding layer, no additional conversion for BN layers is necessary. Empirically we found loss-less conversion when the BN parameters are integrated into other weights. The advantage lies purely in obtaining better performing ANNs if BN is used during training.

#### 2.2.4. Analog input to first hidden layer

Because event-based benchmark datasets are rare (Hu et al., 2016; Rueckauer and Delbruck, 2016), conventional frame-based image databases such as MNIST (LeCun et al., 1998) or CIFAR (Krizhevsky, 2009) have been used to evaluate the classification error rate of the converted SNN. Previous methods (Cao et al., 2015; Diehl et al., 2015) usually transform the analog input activations, e.g., gray levels or RGB values, into Poisson firing rates. But this transformation introduces variability into the firing of the network and impairs its performance.

Here, we interpret the analog input activations as constant currents. Following Equation (2), the input to the neurons in the first hidden layer is obtained by multiplying the corresponding kernels with the analog input image x:

(8) $$\begin{array}{l} z_{i}^{1}:=V_{\text{thr}}\left(\overset{M^{0}}{\underset{j=1}{\sum }}W_{ij}^{1}x_{j}+b_{i}^{1}\right)\;. \end{array}$$

This results in one constant charge value $z_{i}^{l}$ per neuron *i*, which is added to the membrane potential at every time step. The spiking output then begins with the first hidden layer. Empirically we found this to be particularly effective in the low-activation regime of ANN units, where usually undersampling in spiking neurons poses a challenge for successful conversion.

#### 2.2.5. Spiking softmax

Softmax is commonly used on the outputs of a deep ANN, because it results in normalized and strictly positive class likelihoods. Previous approaches for ANN-to-SNN conversion did not convert softmax layers, but simply predicted the output class corresponding to the neuron that spiked most during the presentation of the stimulus. However, this approach fails when all neurons in the final layer receive negative inputs, and thus never spike.

Here we implement two versions of a spiking softmax layer. The first is based on the mechanism proposed in Nessler et al. (2009), where output spikes are triggered by an external Poisson generator with fixed firing rate. The spiking neurons do not fire on their own but simply accumulate their inputs. When the external generator determines that a spike should be produced, a softmax competition according to the accumulated membrane potentials is performed. The second variant of our spiking softmax function is similar, but does not rely on an external clock. To determine if a neuron should spike, we compute the softmax on the membrane potentials, and use the resulting values in range of [0, 1] as rate parameters in a Poisson process for each neuron. In both variants, the final classification result over the course of stimulus presentation is then given by the index of the neuron with the highest firing rate, as before. We prefer the second variant because it does not depend on an additional hyperparameter. A third variant has been suggested by one of the reviewers: Since the softmax is applied at the last layer of the network, one could simply infer the classification output from the softmax computed on the membrane potentials, without another spike generation mechanism. This simplification could speed up inference time and possibly improve the accuracy by reducing stochasticity. This method is appealing where one does not insist upon a purely spiking network.

#### 2.2.6. Spiking max-pooling layers

Most successful ANNs use max-pooling to spatially down-sample feature maps. However, this has not been used in SNNs because computing maxima with spiking neurons is non-trivial. Instead, simple average pooling used in Cao et al. (2015), Diehl et al. (2015), results in weaker ANNs being trained before conversion. Lateral inhibition, as suggested in Cao et al. (2015), does not fulfill the job properly, because it only selects the winner, but not the actual maximum firing rate. Another suggestion is to use a temporal Winner-Take-All based on time-to-first-spike encoding, in which the first neuron to fire is considered the maximally firing one (Masquelier and Thorpe, 2007; Orchard et al., 2015b). Here we propose a simple mechanism for spiking max-pooling, in which output units contain gating functions that only let spikes from the maximally firing neuron pass, while discarding spikes from other neurons. The gating function is controlled by computing estimates of the pre-synaptic firing rates, e.g., by computing an online or exponentially weighted average of these rates. In practice we found several methods to work well, but demonstrate only results using a finite impulse response filter to control the gating function.

### 2.3. Counting operations

To obtain the number of operations in the networks during classification, we define as fan-in *f*_in_ the number of incoming connections to a neuron, and similarly fan-out *f*_out_ as the number of outgoing projections to neurons in the subsequent layer. To give some examples: In a convolutional layer, the fan-in is given by the size of the 2-dimensional convolution kernel multiplied by the number of channels in the previous layer. In a fully-connected layer, the fan-in simply equals the number of neurons in the preceding layer. The fan-out of a neuron in a convolutional layer *l* that is followed by another convolution layer *l* + 1 generally depends on the stride of layer *l* + 1. If the stride is 1, the fan-out is simply given by the size of the 2-dimensional convolution kernel of layer *l* + 1, multiplied by the number of channels in layer *l* + 1. Note that the fan-out may be reduced in corners and along edges of the feature map depending on how much padding is applied.

In case of the ANN, the total number of floating-point operations for classification of one frame is given by:

(9) $$\begin{array}{l} \overset{L}{\underset{l=1}{\sum }}\left(2f_{\text{in},l}+1\right)n_{l}\text{ Ops}/\text{frame}, \end{array}$$

with *n*_*l*_ the number of neurons in layer *l*. The factor 2 comes from the fact that each fan-in operation consist of a multiplication and addition. With +1, we count the operations needed to add the bias. The pooling operation is not considered here.

In the case of an SNN, only additions are needed when the neuron states are updated. We adopt the notation from Merolla et al. (2014) and report the *Synaptic Operations*, i.e., the updates in the neurons of a layer caused by a spike in the previous layer^5^. The total number of synaptic operations in the SNN across the simulation duration *T* is

(10) $$\begin{array}{l} \overset{T}{\underset{t=1}{\sum }}\left[\overset{L}{\underset{l=1}{\sum }}f_{\text{out},l}s_{l}\left(t\right)\right]\text{ Ops}/\text{frame}, \end{array}$$

where *s*_*l*_(*t*) denotes the number of spikes fired in layer *l* at time *t*.

In the ANN, the number of operations needed to classify one image, consisting of the cost of a full forward-pass, is a constant. In the SNN, the image is presented to the network for a certain simulation duration, and the network outputs a classification guess at every time step. By measuring both the classification error rate and the operation count at each step during simulation, we are able to display how the classification error rate of the SNN gradually decreases with increasing number of operations (cf **Figure 4**).

The two different modes of operation—single forward pass in the ANN vs. continuous simulation in the SNN—have significant implications when aiming for an efficient hardware implementation. One well known fact is that additions required in SNNs are cheaper than multiply accumulates needed in ANNs. For instance, our simulations in a Global Foundry 28 nm process show that the cost of performing a 32-bit floating-point addition is about 14 X lower than that of a MAC operation and the corresponding chip area is reduced by 21 X. It has also been shown that memory transfer outweighs the energy cost of computations by two orders of magnitude (Horowitz, 2014). In the ANN, reading weight kernels and neuron states from memory, and writing states back to memory is only done once during the forward pass of one sample. In contrast, memory access in the SNN is less predictable and has to be repeated for individual neurons in proportion to their spike rates. If the number of operations needed by the SNN to achieve a similar classification error as that of the ANN is lower, then equivalently the SNN would also have a reduction in the number of memory accesses. The direct implementation of SNNs on dedicated spiking hardware platforms like SpiNNaker or TrueNorth is left to future work, and will be necessary for estimating the real energy cost in comparison to the cost of implementing the original ANNs on custom ANN hardware accelerators like Eyeriss (Chen et al., 2017).

## 3. Results

There are two ways of improving the classification error rate of an SNN obtained via conversion: (1) training a better ANN before conversion, and (2) improving the conversion by eliminating approximation errors of the SNN. We proposed several techniques for these two approaches in section 2; in sections 3.1 and 3.2 we evaluate their effect using the CIFAR-10 data set. section 3.3 extends the SNN conversion methods to the ImageNet data set. In section 3.4 we show that SNNs feature an accuracy-vs.-operations trade-off that allow tuning the performance of a network to a given computational budget.

The networks were implemented in Keras (Chollet, 2015). Some of the CIFAR-10 results were previously reported in Rueckauer et al. (2016).

### 3.1. Contribution of improved ANN architectures

The methods introduced in section 2 allow conversion of CNNs that use biases, softmax, batch-normalization, and max-pooling layers, which all improve the classification error rate of the ANN. The performance of a converted network was quantified on the CIFAR-10 benchmark (Krizhevsky, 2009), using a CNN with 4 convolution layers (32 3×3 - 32 3×3 - 64 3×3 - 64 3×3), ReLU activations, batch-normalization, 2×2 max-pooling layers after the 2nd and 4th convolutions, followed by 2 fully connected layers (512 and 10 neurons respectively) and a softmax output. This ANN achieved 12.14% error rate (Table 1). Constraining the biases to zero increased the error rate to 12.27%. Replacing max-pooling by average-pooling further decreased the performance to 12.31%. Eliminating the softmax and using only ReLUs in the output led to a big drop to 30.56%. With our new methods we can therefore start the conversion already with much better ANNs than was previously possible.

**Table 1:** Classification error rate on MNIST, CIFAR-10 and ImageNet for our converted spiking models, compared to the original ANNs, and compared to spiking networks from other groups.

**Data set [architecture]**	**ANN err**.	**SNN err**.	**Neur**.	**Synap**.
MNIST [ours]	0.56	**0.56**	8 k	1.2 M
MNIST [Zambrano and Bohte, 2016]	0.86	0.86	27 k	6.6 M
CIFAR-10 [ours, BinaryNet sign]	11.03	11.75	0.5 M	164 M
CIFAR-10 [ours, BinaryNet Heav]	11.58	12.55	0.5 M	164 M
CIFAR-10 [ours, BinaryConnect, binarized at infer.]	16.81	16.65	0.5 M	164 M
CIFAR-10 [ours, BinaryConnect, full prec. at infer.]	8.09	**9.15**	0.5 M	164 M
CIFAR-10 [ours]	11.13	11.18	0.1 M	23 M
CIFAR-10 [Esser et al., 2016], 8 chips	NA	12.50	8 M	NA
CIFAR-10 [Esser et al., 2016], single chip	NA	17.50	1 M	NA
CIFAR-10 [Hunsberger and Eliasmith, 2016]^*^	14.03	16.46	50 k	NA
CIFAR-10 [Cao et al., 2015]^**^	20.88	22.57	35 k	7.4 M
ImageNet [ours, VGG-16]^†^	36.11 (15.14)	50.39 (18.37)	15 M	3.5 B
ImageNet [ours, Inception-V3]^††^	23.88 (7.01)	**25.40** (**7.96**)	11.7 M	0.5 B
ImageNet [Hunsberger and Eliasmith, 2016]^‡^	NA	48.20 (23.80)	0.5 M	NA

*The reported error rate is top-1, with top-5 in brackets for ImageNet*.

* *Cropped to 24x24*.

** *Cropped to 24x24*.

† *On a subset of 2570 samples, using single-scale images of size 224x224*.

†† *On a subset of 1382 samples, using single-scale images of size 299x299*.

‡ *On a subset of 3072 samples. The values in bold highlight the best SNN result for a particular data set*.

### 3.2. Contribution of improved SNN conversion methods

**Figure 2:**
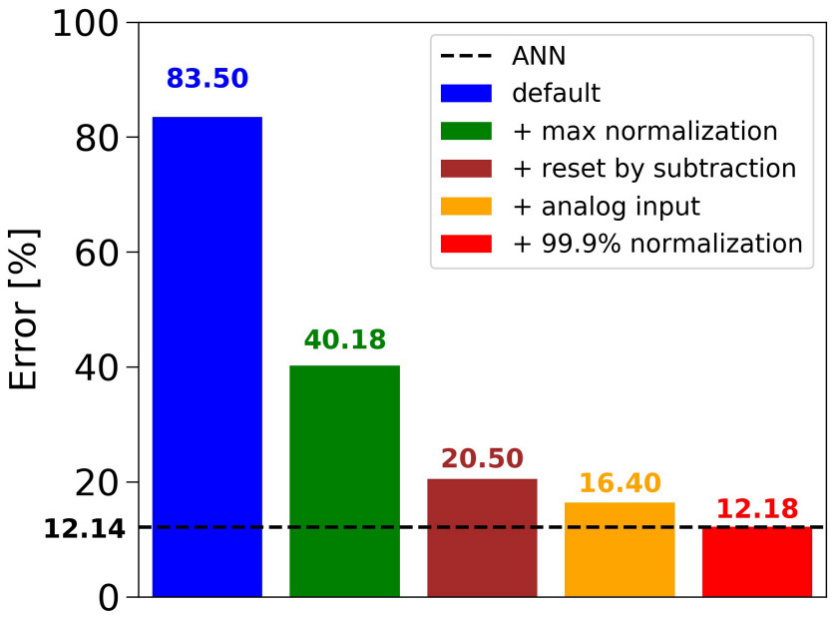
Influence of novel mechanisms for ANN-to-SNN conversion on the SNN error rate for CIFAR-10.

Figure 2 shows that in the case of CIFAR-10, the conversion of the best ANN into an SNN using the default approach (i.e., no normalization, Poisson spike train input, reset-to-zero) fails, yielding an error rate of 83.50%, barely above chance level. Adding the data-based weight normalization (Diehl et al., 2015) (green bar) lowers the error rate to 40.18%, but this is still a big drop from the ANN result of 12.14% (dashed black line). Changing to the *reset-by-subtraction* mechanism from section 2.1 leads to another 20% improvement (brown bar), and switching to analog inputs to the first hidden layer instead of Poisson spike trains results in an error rate of 16.40% (orange bar). Finally, using the 99.9th percentile of activations for robust weight normalization yields 12.18% error rate, which is on par with the ANN performance and gives our best result for CIFAR-10. We therefore conclude that our proposed mechanisms for ANN training and ANN-to-SNN conversion contribute positively to the success of the method. The conversion into a SNN is nearly loss-less, and the results are very competitive for classification benchmarks using SNNs (Table 1). These results were confirmed also on MNIST, where a 7-layer network with max-pooling achieved an error rate of 0.56%, thereby improving previous state-of-the-art results for SNNs reported by Diehl et al. (2015) and Zambrano and Bohte (2016).

**Figure 3:**
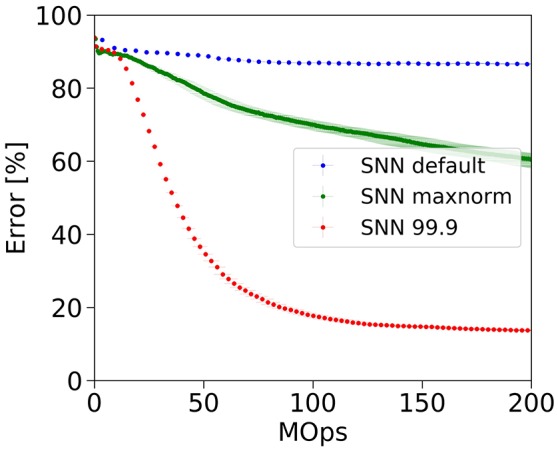
Accuracy-latency trade-off. Robust parameter normalization (red) enables our spiking network to correctly classify CIFAR-10 samples much faster than using our previous max-normalization (green). Not normalizing leads to classification at chance level (blue).

SNNs are known to exhibit a so-called accuracy-latency trade-off (Diehl et al., 2015; Neil et al., 2016), which means that the error rate drops the longer the network is simulated, i.e., the more operations we invest. The latency in which the final error rate is achieved, is dependent on the type of parameter normalization as illustrated by the three curves in Figure 3. Parameter normalization is necessary to improve upon chance-level classification (blue, no normalization). However, our previous max-norm method (green) converges very slowly to the ANN error rate because the weight scale is overly reduced and spike-activity is low. With a robust normalization using the 99.9th percentile of the activity distribution, the weights are larger and convergence is much faster. Empirically, the best results were obtained with normalization factors in the range between the 99th and 99.9th percentile of activations, which allows the network to converge quickly to error rates similar to those of the underlying ANN.

This accuracy-latency trade-off is very prominent in case of the classic LeNet architecture on MNIST (**Figure 5**). While the ANN achieves an error rate of 1.04 % using a fixed amount of 2.35 MOps per frame, the spiking model reaches within 1 percentage point of the ANN using 2x less operations (2.07 % error rate at 1.07 MOps/frame). At 1.47 MOps, the SNN error rate is 1.13 %. The SNN then continues to improve until it reaches 1.07 % error rate at the end of the simulation.

### 3.3. ImageNet

VGG Simonyan and Zisserman (2014) and GoogLeNet Szegedy et al. (2015) are two deep network architectures that won first places in the localization and classification competitions of the ImageNet ILSVRC-2014 respectively. By introducing *inception modules* and *bottlenecks*, GoogLeNet requires 12X fewer parameters and significantly less computes than VGG-16, even though the total layer count is much higher. Since their initial introduction in 2014, both architectures have been improved. The third version of GoogLeNet which was released in 2015 as Inception-V3 (Szegedy et al., 2016), improved on the ImageNet results to state-of-the art 5.6% top-5 error rate, and uses 2.5X more computes than the original GoogLeNet. This was in part done by further reducing the kernel size and dimensions inside the network, applying regularization via batch-normalized auxiliary classifiers, and label smoothing.

#### 3.3.1. Transient dynamics and voltage clamp

While the conversion pipeline outlined in section 2 can deliver converted SNNs that produced equivalent error rates as the original ANNs on the MNIST and CIFAR-10 data sets, the error rate of the converted Inception-V3 was initially far from the error rate of the ANN. One main reason is that neurons undergo a transient phase at the beginning of the simulation because a few neurons have large biases or large input weights. During the first few time steps, the membrane potential of each neuron needs to accumulate input spikes before it can produce any output. The firing rates of neurons in the first layer need several time steps to converge to a steady rate, and this convergence time is increased in higher layers that receive transiently varying input. The convergence time is decreased in neurons that integrate high-frequency input, but increased in neurons integrating spikes at low frequency^6^. Another factor contributing to a large transient response are 1 × 1 convolution layers. In these layers, the synaptic input to a single neuron consists only of a single column through the channel-dimension of the previous layer, so that the neuron's bias or a single strongly deviating synaptic input may determine the output dynamics. With larger kernels, more spikes are gathered that can outweigh the influence of e.g., a large bias^7^.

In order to overcome the negative effects of transients in neuron dynamics, we tried a number of possible solutions, including the initializations of the neuron states, different reset mechanisms, and bias relaxation schemes. The most successful approach we found was to clamp the membrane potential to zero for the first *N* time-steps, where *N* increases linearly with the layer depth *l*: *N*(*l*) = *d* · *l*. The slope *d* represents the temporal delay between lifting the clamp from consecutive layers. The longer the delay *d*, the more time is given to a previous layer to converge to steady-state before the next layer starts integrating its output.

This simple modification of the SNN state variables removes the transient response completely (see Figure S1), because by the time the clamp is lifted from post-synaptic neurons, the presynaptic neurons have settled at their steady-state firing-rate. We found a clamping delay of *d* = 10 in Inception-V3 to be sufficient. Clamping the membrane potential in VGG-16 did not have a notable impact on the error rate. Each input image was presented to the converted VGG-16 spiking network for 400 time steps, and to the converted Inception-V3 for 550 time steps. The average firing rate of neurons is 0.016 Hz^8^ in VGG-16, and 0.053 Hz in Inception-V3.

We expect that the transient of the network could be reduced by training the network with constraints on the biases or the β parameter of the batch-normalization layers. Table 1 summarizes the error rates achieved by our SNNs using the methods presented above, and compares them to previous work by other groups.

### 3.4. Combination with low-precision models

The neurons in our spiking network emit events at a rate proportional to the activation of the corresponding unit in the ANN. Target activations with reduced precision can be approximated more quickly and accurately with a small number of spike events. For instance, if the activations are quantized into values of {0, 0.1, 0.2, …, 0.9, 1.0}, the spiking neuron can perfectly represent each value within at most 10 time steps. On the other hand, to approximate a floating-point precision number using 16 bit precision, the neuron in the worst case would have to be active for 2^16^ = 65536 time steps.

To demonstrate the potential benefit of using low-precision activations when transforming a given model into a spiking network, we apply the methods from section 2.2 to BinaryNet Courbariaux et al. (2016), a CNN where both weights and activations are constrained to either {0, +1}, or {−1, +1}. To obtain the binarized ANNs with these two sets of activations, we train BinaryNet using the publicly available source code Courbariaux et al. (2016) on two different activation functions: First with a Heaviside activation function, and second, with a signed activation function. The two binarized models are then converted into spiking networks. Instead of interpreting the negative activations of BinaryNet “sign” as negative firing rates, we invert the sign of the spikes emitted by neurons with a negative activation. To achieve this, we add a second threshold at −1, where neurons can emit spikes of size −1 if the threshold is reached from above.

**Figure 4:**
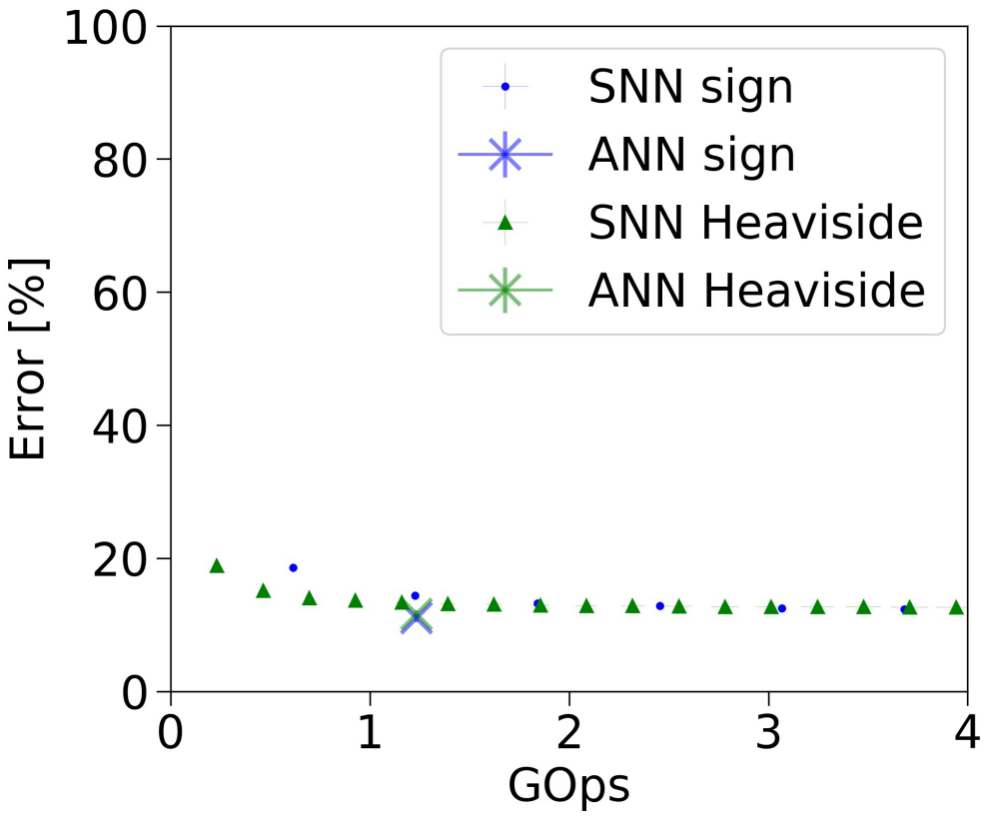
Classification error rate vs number of operations for the BinaryNet ANN and SNN implementation on the complete CIFAR-10 dataset.

**Figure 5:**
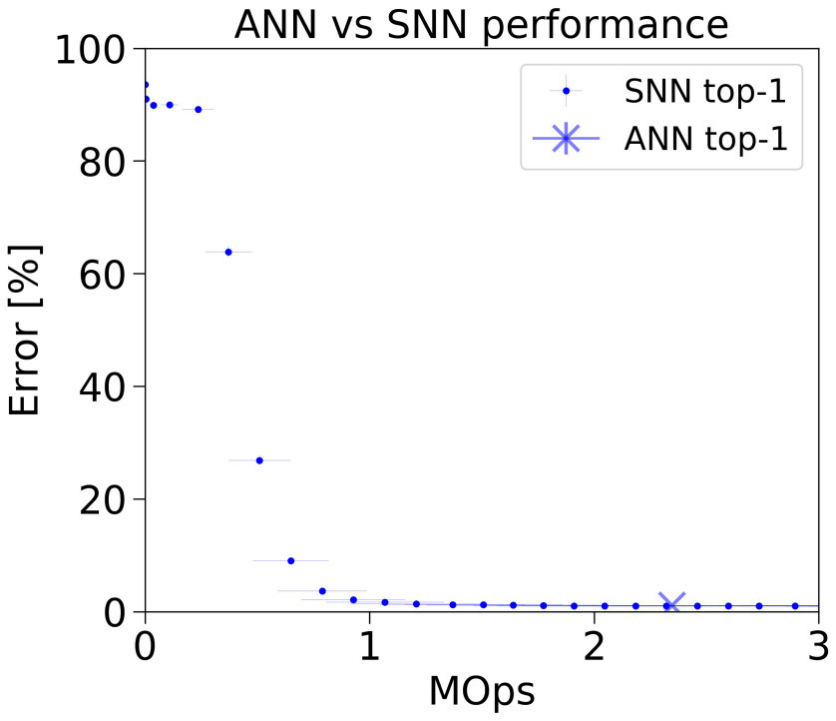
Classification error rate vs number of operations for the LeNet ANN and SNN implementation on the MNIST dataset.

By virtue of the quantized activations, these two SNNs are able to approximate the ANN activations with very few operations (see Figure 4). The BinaryNet SNNs already show an error rate which is close to the ANN target error rates early in the simulation, in fact as soon as the first output spikes are produced. In contrast, in full-precision models (cf. Figures 3, 5), the classification error rate starts at chance level and drops over the course of the simulation, as more operations are invested.

The lowest error rate for our converted spiking CIFAR-10 models is achieved using BinaryConnect (Courbariaux et al., 2015). This network is trained using full-precision weights in combination with binarized weights. Either set of weights can be used during inference. We test the resulting model with both the binarized weights and the full-precision copy kept during training (cf. Table 1). These results illustrate how spiking networks benefit from and at the same time complement the strengths of low-precision models.

## 4. Discussion

This work presents two new developments. The first is a novel theory that describes the approximation of an SNN firing rates to its equivalent ANN activations. The second is the techniques to convert almost arbitrary continuous-valued CNNs into spiking equivalents. By implementing SNN-compatible versions of common ANN CNN features such as max pooling, softmax, batch normalization, biases and Inception modules, we allow a larger class of CNNs including VGG-16 and GoogLeNet Inception-V3 to be converted into SNNs. Table 1 shows that our SNN results compare favorably to previous SNN results on all tested data sets: (Cao et al., 2015) achieved 22.57% error rate on CIFAR-10, albeit with a smaller network and after cropping images to 24×24. With a similarly small network and cropped images, (Hunsberger and Eliasmith, 2016) achieve 16.46% error rate. Better SNN error rates to date have only been reported by Esser et al. (2016), where an error rate of 12.50% was reported for a very large network optimized for 8 TrueNorth chips, and making use of ternary weights and multiple 1×1 network-in-network layers. A smaller network fitting on a single chip is reported to achieve 17.50%. In our own experiments with similar low-precision training schemes for SNNs, we converted the BinaryConnect model by Courbariaux et al. (2016) to 8.65% error rate on CIFAR10, which is by far the best SNN result reported to date.

In addition to the improved SNN results on MNIST and CIFAR-10, this work presents for the first time, a spiking network implementation of VGG-16 and Inception-V3 models, utilizing simple non-leaky integrate-and-fire neurons. The top-5 error rates of the SNNs during inference lie close to the original ANNs. Future investigations will be carried out to identify additional conversion methods that will allow the VGG-16 SNN to reach the error rate of the ANN. For instance, we expect a reduction in the observed initial transients of higher up layers within large networks, by training the networks with constraints on the biases.

With BinaryNet (an 8-layer CNN with binary weights and activations tested on CIFAR-10) (Courbariaux et al., 2016), we demonstrated that low-precision models are well suited for conversion to spiking networks. While the original network requires a fixed amount of 1.23 GOps to classify a single frame with average error rate of 11.57%, the SNN can be queried for a classification result at a variable number of operations. For instance, the average error rate of the SNN is 15.13% at 0.46 GOps (2.7x reduction), and improves further when investing more operations. This reduction in operation count is due to the fact that, first, activation values at lower precision can more easily be approximated by discrete spikes, and second, zero activations are natively skipped in the activity-driven operation of spiking networks. In light of this, our work builds upon and complements the recent advances in low-precision models and network compression.

The converted networks highlight a remarkable feature of spiking networks: While ANNs require a fixed amount of computations to achieve a classification result, the final error rate in a spiking network drops off rapidly during inference when an increasing number of operations is used to classify a sample. The network classification error rate can be tailored to the number of operations that are available during inference, allowing for accurate classification at low latency and on hardware systems with limited computational resources. In some cases, the number of operations needed for correct classification can be reduced significantly compared to the original ANN. We found a savings in computes of 2x for smaller full-precision networks (e.g., LeNet has 8 k neurons and 1.2 M connections), and larger low-precision models (e.g., BinaryNet has 0.5 M neurons and 164 M connections). These savings did not scale up to the very large networks such as VGG-16 and Inception-V3 with more than 11 M neurons and over 500 M connections. One reason is that each additional layer in the SNN introduces another stage where high-precision activations need to be approximated by discrete spikes. We show in Equation (5b) that this error vanishes over time. But since higher layers are driven by inputs that contain approximation errors from lower layers (cf. Equation 6), networks of increasing depth need to be simulated longer for an accurate approximation. We are currently investigating spike encoding schemes that make more efficient use of temporal structure than the present rate-based encoding. Mostafa et al. (2017) present such an approach where the precise spike time is used to train a network to classify MNIST digits with a single spike per neuron. Such a sparse temporal code clearly reduces the cost of repeated weight fetches which dominates in rate-encoded SNNs.

Finally, this conversion framework allows the deployment of state-of-the-art pre-trained high-performing ANN models onto energy-efficient real-time neuromorphic spiking hardware such as TrueNorth (Benjamin et al., 2014; Merolla et al., 2014; Pedroni et al., 2016).

## Author contributions

BR developed the theory, implemented the methods, conducted the experiments and drafted the manuscript. YH implemented and tested the spiking max-pool layer. I-AL contributed to some of the experiments. MP and S-CL contributed to the design of the experiments, the analysis of the data, and to the writing of the manuscript.

### Conflict of interest statement

The authors declare that the research was conducted in the absence of any commercial or financial relationships that could be construed as a potential conflict of interest. The reviewer, SS, and handling Editor declared their shared affiliation.

## Footnotes

^1^https://github.com/BVLC/caffe/wiki/Model-Zoo

^2^http://snntoolbox.readthedocs.io/

^3^This analysis focuses on applications with image data sets, which are generally transformed in this way. The argument could be extended to the case of zero-centered data by interpreting negative input to the first hidden layer of the SNN as coming from a class of inhibitory neurons, and inverting the sign of the charge deposited in the post-synaptic neuron.

^4^This distribution is obtained by computing the ANN activations on a large fraction of the training set. From this, the scaling factor can be determined and applied to the layer parameters. This has to be done only once for a given network; during inference the parameters do not change.

^5^This *synaptic* operation count does not include updates of the state variables due to a bias or dynamics of the post-synaptic potential (which is instantaneous in our case). We validated in our simulations that the operations caused by the bias are about two orders of magnitude fewer in number than synaptic operations, in addition to being less costly in terms of memory fetches).

^6^An ANN neuron responds precisely the same whether (A) receiving input from a neuron with activation 0.1 and connecting weight 0.8, or (B) activation 0.8 and weight 0.1. In contrast, the rate of an SNN neuron will take longer to converge in case (A) than in (B). This phenomenon forms the basis of the accuracy-latency trade-off mentioned above: One would like to keep firing rates as low as possible to reduce the operational cost of the network, but has to sacrifice approximation accuracy for it.

^7^Even though the parameters in each layer were normalized such that the input to each neuron is below threshold, this does not guarantee that all biases are sub-threshold: their effect could be reduced by inhibitory input spikes. While such inhibitory synaptic input is still missing at the onset of the simulation, the output dynamics of a neuron will be dominated by a large bias.

^8^As our neuron model does not contain any time constant, this unit should be read as “spikes per simulation time step” and is not related to spikes per wall-clock time.
