# Supplementary material for: Conversion of Continuous-Valued Deep Networks to Efficient Event-Driven Networks for Image Classification
Source: Front Neurosci. 2017 Dec 7;11:682. doi: 10.3389/fnins.2017.00682 (PMC5770641; doi:10.3389/fnins.2017.00682)
Supplement: Supplementary file 1 [file Image1.pdf]

Supplementary Material: Conversion of Continuous-Valued Deep Networks to Efficient Event-Driven Networks for Image Classification

Bodo Rueckauer [1,*], Iulia-Alexandra Lungu [1], Yuhuang Hu [1], Michael Pfeiffer [1,2], and Shih-Chii Liu [1]
*Correspondence:
Bodo Rueckauer, Institute of Neuroinformatics, University of Zurich and ETH Zurich, Winterthurerstrasse 190, 8057 Zurich, Switzerland
rbodo@ini.uzh.ch

1 RELATION BETWEEN SNN FIRING RATES AND ANN ACTIVATIONS

1.1 Reset to zero

Our goal is to derive a relation between the firing rate $r_i^1(t)$ of a neuron i in layer 1 of the SNN and the activation of the corresponding neuron in the ANN. To simplify the notation, we drop the layer and neuron indices. Starting from the membrane equation (4a), the average firing rate can simply be computed by summing over the simulation time t:

$$\sum_{t'=1}^{t} V(t') = \sum_{t'=1}^{t} \big(V(t'-1) + z\big)\,(1 - \Theta_{t'}) \tag{S1}$$

Under the assumption of constant analog input z to the first hidden layer (cf. Sec. 2.2.4), and using $N(t) := \sum_{t'=1}^{t} \Theta_{t'}$ we obtain

$$\sum_{t'=1}^{t} V(t') = \sum_{t'=1}^{t} V(t'-1)(1 - \Theta_{t'}) + z\left(\frac{t}{\Delta t} - N\right). \tag{S2}$$

The time resolution Δt enters the equation when evaluating the time-sum over a constant: $\sum_{t'}^{t} 1 = t/\Delta t$. It will be replaced by the definition of the maximum firing rate $r_{\text{max}} = 1/\Delta t$ in the following.

By rearranging Equation (S2) to yield the total number of spikes N, and dividing by the simulation time t we obtain the average firing rate r of a neuron in layer 1:

$$r := \frac{N}{t} = r_{\text{max}} - \frac{1}{zt}\sum_{t'=1}^{t}(V(t') - V(t'-1)(1 - \Theta_{t'})). \tag{S3}$$

By rearranging the indices in the sum on the right-hand side, equation (S3) simplifies to

$$r = r_{\max} - \frac{V(t) - V(0)}{zt} - \frac{1}{zt}\sum_{t'=1}^{t} V(t'-1)\Theta_{t'}. \tag{S4}$$

Since Θ_t equals 1 if there is a spike and 0 otherwise, the last term in equation (S4) sums up the membrane potentials of the neuron just before a spike. In the case of reset-to-zero, constant input, constant threshold, and no leak, the value of the membrane potential immediately before a spike is always the same, and is always an integer multiple of the input z. Let therefore $n \in \mathbb{N}$ be the number of time-steps needed to cross threshold, i.e. $(n-1)z < V_{\text{thr}} \leq nz$. Then

$$\frac{1}{zt}\sum_{t'=1}^{t} V(t'-1)\Theta_{t'} = \frac{1}{zt}(n-1)zN = (n-1)r. \tag{S5}$$

With this, the expression for the firing rate (S3) becomes

$$r = \frac{1}{n}\left(r_{\max} - \frac{V(t) - V(0)}{zt}\right). \tag{S6}$$

We now define the residual $\epsilon \in \mathbb{R}$ as the surplus charge above threshold at the time of spike:

$$\epsilon := nz - V_{\text{thr}}. \tag{S7}$$

Plugging this into equation (S6), the average spike rate is given by

$$r = \frac{z}{V_{\text{thr}} + \epsilon}\left(r_{\max} - \frac{V(t) - V(0)}{zt}\right). \tag{S8}$$

Lastly, we make use of the fact that in the first hidden layer at constant input, $z^1 = V_{\text{thr}}a^1$. Setting $V(0) = 0$, reordering the terms, and reintroducing the dropped indices yields Equation (5a):

$$r_i^1(t) = a_i^1 r_{\max}\frac{V_{\text{thr}}}{V_{\text{thr}} + \epsilon_i^1} - \frac{V_i^1(t)}{t(V_{\text{thr}} + \epsilon_i^l)}. \tag{S9}$$

1.2 Reset by subtraction

The derivation of a relation between rates r and activations a simplifies greatly for the case of *reset by subtraction*. Averaging the membrane equation (4b) over the simulation time t yields:

$$\frac{1}{t}\sum_{t'=1}^{t} V(t') = \frac{1}{t}\sum_{t'=1}^{t} V(t'-1) + zr_{\max} - V_{\text{thr}}\frac{N}{t}. \tag{S10}$$

Using $z^1 = V_{\text{thr}}a^1$ and solving for $r = N/t$ leads to

$$r(t) = ar_{\max} - \frac{1}{V_{\text{thr}}t}\sum_{t'=1}^{t}(V(t') - V(t'-1)) = ar_{\max} - \frac{V(t) - V(0)}{V_{\text{thr}}t}. \tag{S11}$$

Setting $V(0) = 0$ and reintroducing the indices yields Equation (5b).

2 TRANSIENT DYNAMICS AND VOLTAGE CLAMP

Figure S1 illustrates how transients manifest themselves as offsets in the spike rates of individual feature maps, and how the voltage clamp technique resolves the problem.

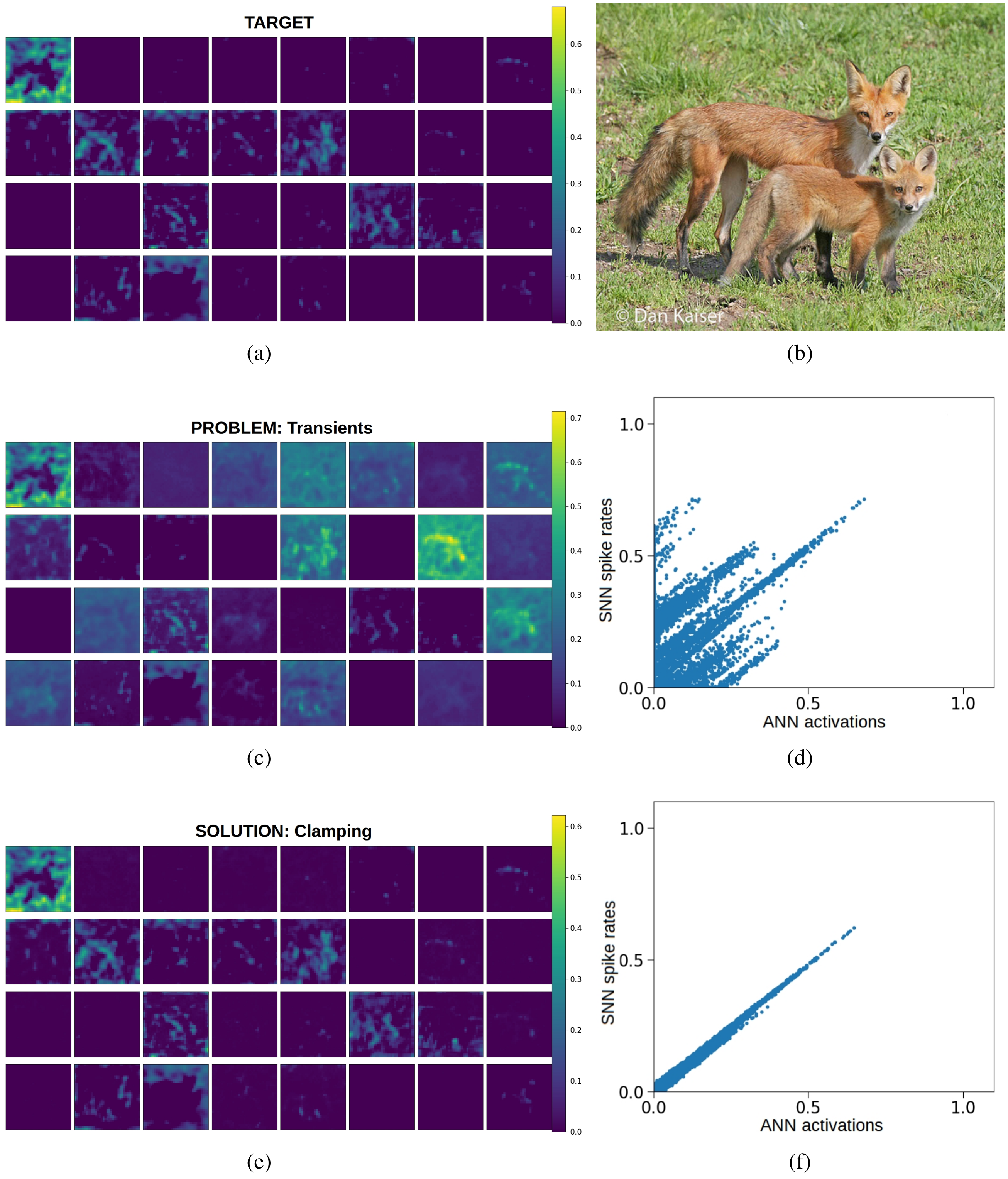

Figure S1: **(a)** Inception-V3 activations in layer 14, consisting of 32 1x1 convolutions. This heat map represents the target rates which the SNN approximates. **(b)** The input image. **(c)** SNN spike-rates of the same layer, when the membrane potential of the SNN is not clamped at the start. Clearly, some feature maps are more active than their corresponding ANN feature maps, other maps are less active. The consistent offset across a feature map points to the bias as the plausible cause for this offset. **(d)** Correlation between ANN activations from (a) and SNN spike rates from (c) reveal the offsets of entire feature maps. **(e)** SNN spike rates of the same layer, with membrane potentials clamped for 10 time steps between consecutive layers. The resulting spike rates are much closer to the ANN target activations. **(f)** ANN-SNN correlation is close to perfect.
